# Supplementary material for: Aminophylline shortage and current recommendations for reversal of vasodilator stress: An ASNC information statement endorsed by SCMR
Source: J Nucl Cardiol. 2018 Dec 20;26(3):1007–14. doi: 10.1007/s12350-018-01548-0 (PMC6517353; doi:10.1007/s12350-018-01548-0)
Supplement: Supplementary file 1 — Supplementary material 1 (PPTX 128 kb) [file 12350_2018_1548_MOESM1_ESM.pptx]

## Slide 1
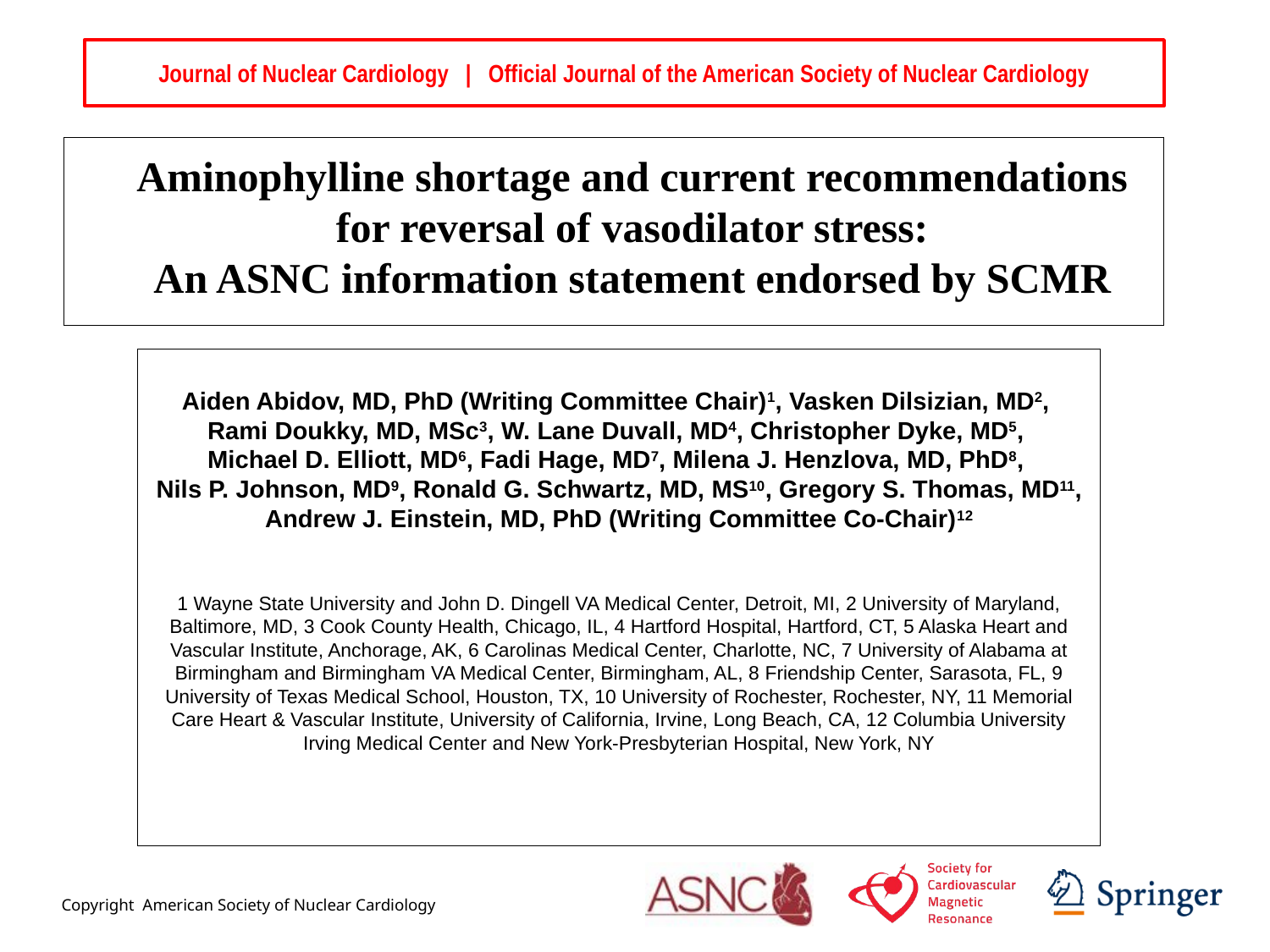

Journal of Nuclear Cardiology | Official Journal of the American Society of Nuclear Cardiology
# Aminophylline shortage and current recommendations for reversal of vasodilator stress: An ASNC information statement endorsed by SCMR
Aiden Abidov, MD, PhD (Writing Committee Chair)1, Vasken Dilsizian, MD2,
Rami Doukky, MD, MSc3, W. Lane Duvall, MD4, Christopher Dyke, MD5,
Michael D. Elliott, MD6, Fadi Hage, MD7, Milena J. Henzlova, MD, PhD8,
Nils P. Johnson, MD9, Ronald G. Schwartz, MD, MS10, Gregory S. Thomas, MD11, Andrew J. Einstein, MD, PhD (Writing Committee Co-Chair)12
1 Wayne State University and John D. Dingell VA Medical Center, Detroit, MI, 2 University of Maryland, Baltimore, MD, 3 Cook County Health, Chicago, IL, 4 Hartford Hospital, Hartford, CT, 5 Alaska Heart and Vascular Institute, Anchorage, AK, 6 Carolinas Medical Center, Charlotte, NC, 7 University of Alabama at Birmingham and Birmingham VA Medical Center, Birmingham, AL, 8 Friendship Center, Sarasota, FL, 9 University of Texas Medical School, Houston, TX, 10 University of Rochester, Rochester, NY, 11 Memorial Care Heart & Vascular Institute, University of California, Irvine, Long Beach, CA, 12 Columbia University Irving Medical Center and New York-Presbyterian Hospital, New York, NY
Copyright American Society of Nuclear Cardiology

## Slide 2
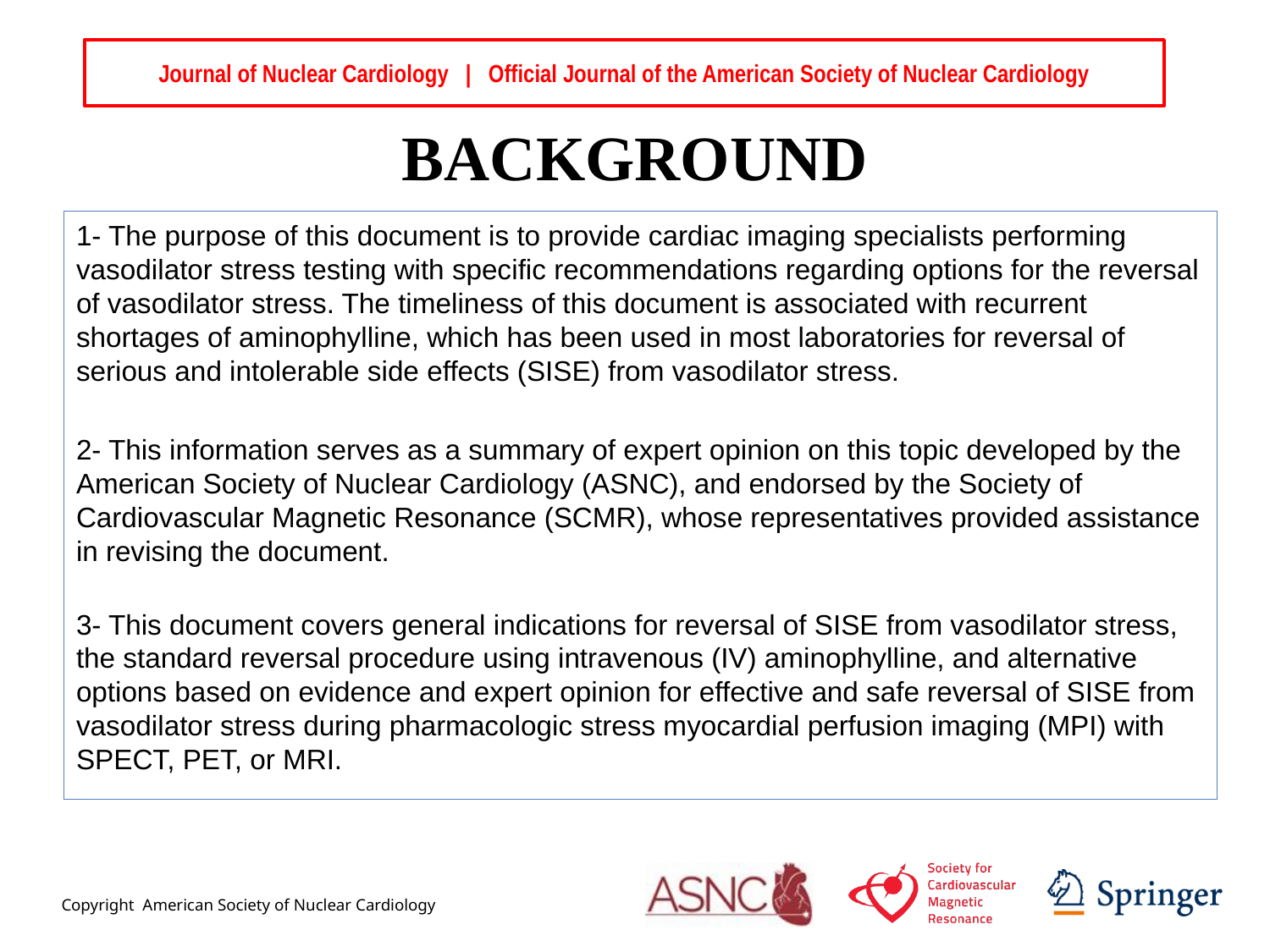

Journal of Nuclear Cardiology | Official Journal of the American Society of Nuclear Cardiology
# BACKGROUND
1- The purpose of this document is to provide cardiac imaging specialists performing vasodilator stress testing with specific recommendations regarding options for the reversal of vasodilator stress. The timeliness of this document is associated with recurrent shortages of aminophylline, which has been used in most laboratories for reversal of serious and intolerable side effects (SISE) from vasodilator stress.
2- This information serves as a summary of expert opinion on this topic developed by the American Society of Nuclear Cardiology (ASNC), and endorsed by the Society of Cardiovascular Magnetic Resonance (SCMR), whose representatives provided assistance in revising the document.
3- This document covers general indications for reversal of SISE from vasodilator stress, the standard reversal procedure using intravenous (IV) aminophylline, and alternative options based on evidence and expert opinion for effective and safe reversal of SISE from vasodilator stress during pharmacologic stress myocardial perfusion imaging (MPI) with SPECT, PET, or MRI.
Copyright American Society of Nuclear Cardiology

## Slide 3
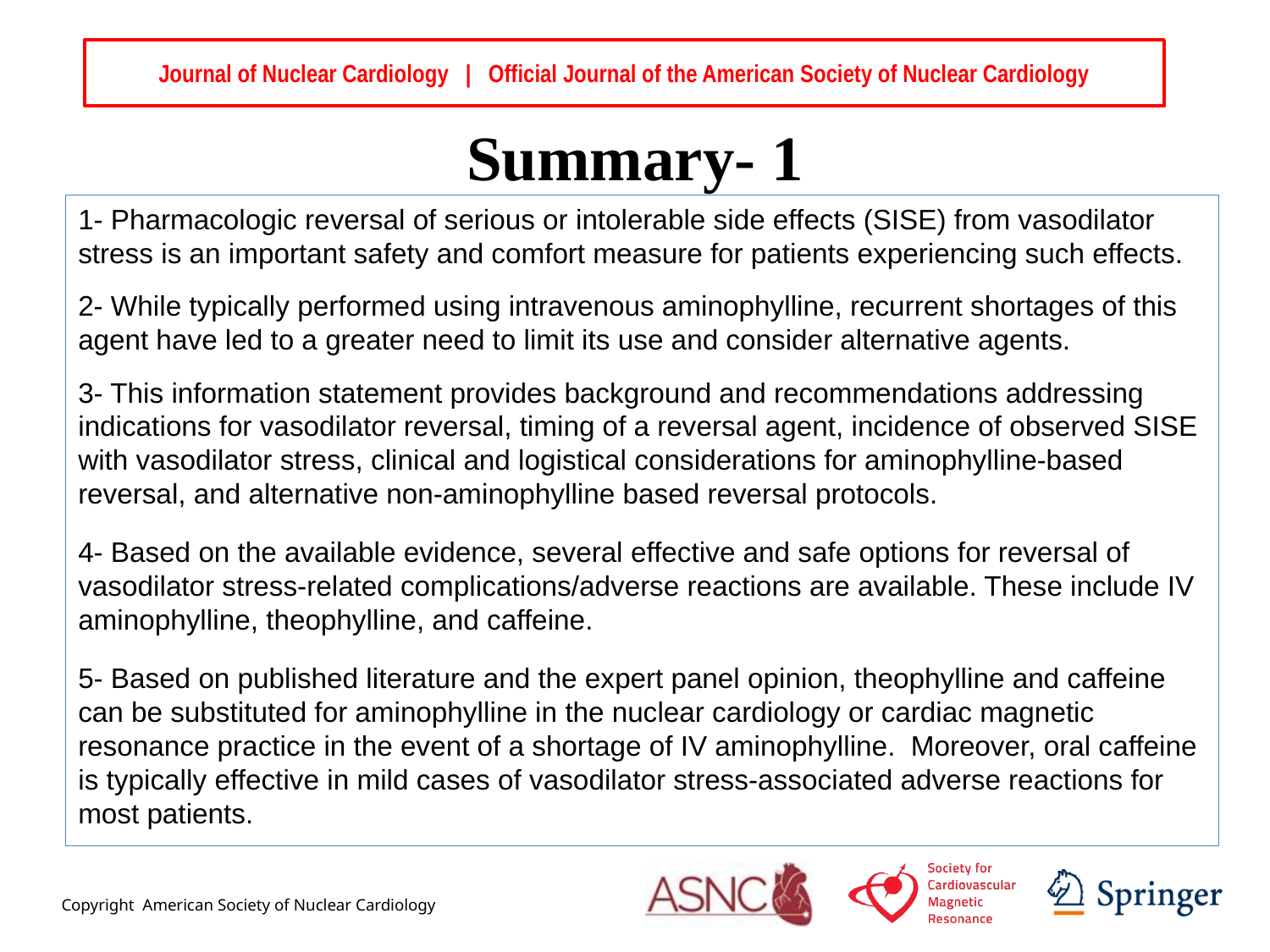

Journal of Nuclear Cardiology | Official Journal of the American Society of Nuclear Cardiology
# Summary- 1
1- Pharmacologic reversal of serious or intolerable side effects (SISE) from vasodilator stress is an important safety and comfort measure for patients experiencing such effects.
2- While typically performed using intravenous aminophylline, recurrent shortages of this agent have led to a greater need to limit its use and consider alternative agents.
3- This information statement provides background and recommendations addressing indications for vasodilator reversal, timing of a reversal agent, incidence of observed SISE with vasodilator stress, clinical and logistical considerations for aminophylline-based reversal, and alternative non-aminophylline based reversal protocols.
4- Based on the available evidence, several effective and safe options for reversal of vasodilator stress-related complications/adverse reactions are available. These include IV aminophylline, theophylline, and caffeine.
5- Based on published literature and the expert panel opinion, theophylline and caffeine can be substituted for aminophylline in the nuclear cardiology or cardiac magnetic resonance practice in the event of a shortage of IV aminophylline. Moreover, oral caffeine is typically effective in mild cases of vasodilator stress-associated adverse reactions for most patients.
Copyright American Society of Nuclear Cardiology

## Slide 4
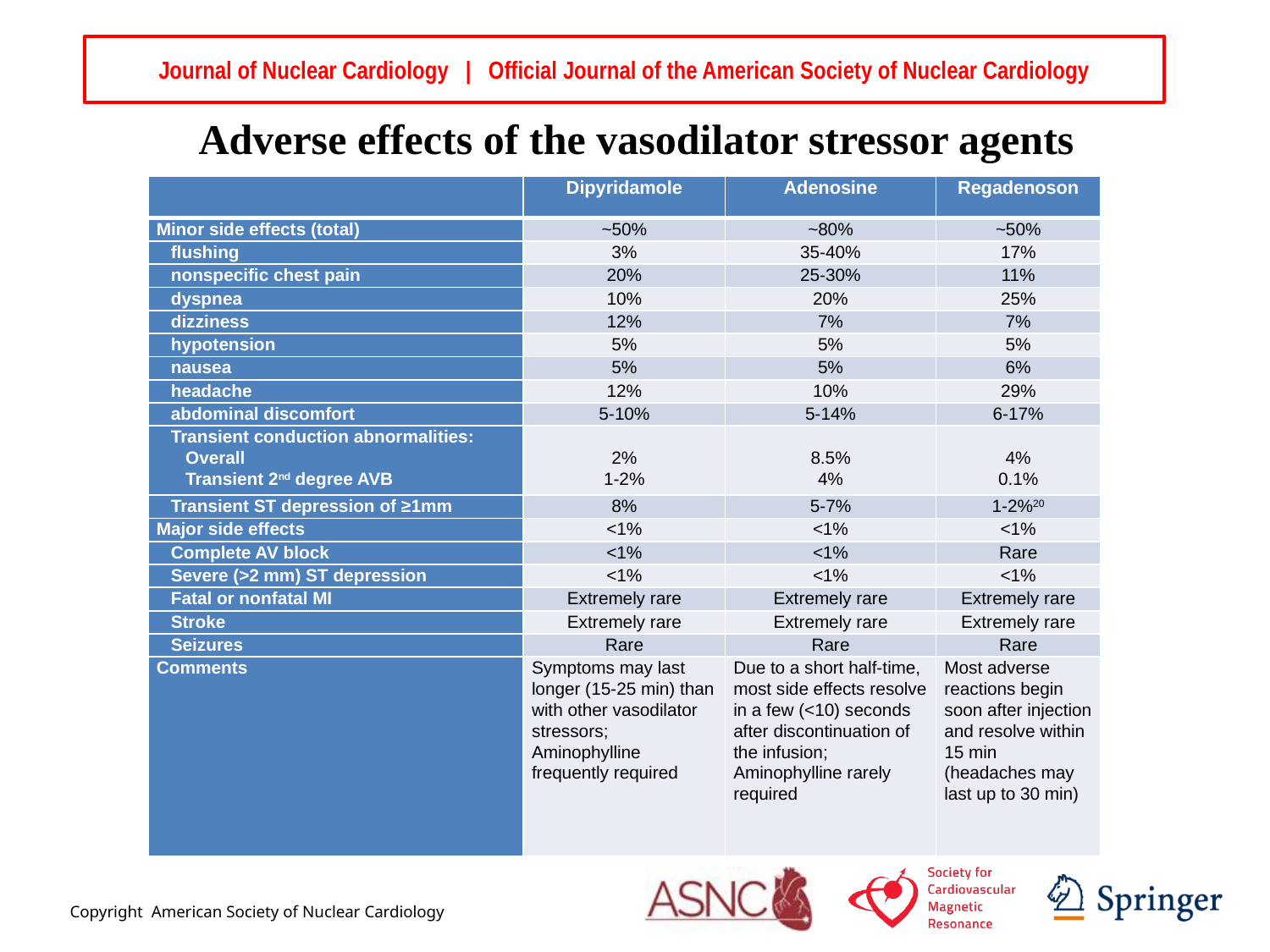

Journal of Nuclear Cardiology | Official Journal of the American Society of Nuclear Cardiology
# Adverse effects of the vasodilator stressor agents
| | Dipyridamole | Adenosine | Regadenoson |
| --- | --- | --- | --- |
| Minor side effects (total) | ~50% | ~80% | ~50% |
| flushing | 3% | 35-40% | 17% |
| nonspecific chest pain | 20% | 25-30% | 11% |
| dyspnea | 10% | 20% | 25% |
| dizziness | 12% | 7% | 7% |
| hypotension | 5% | 5% | 5% |
| nausea | 5% | 5% | 6% |
| headache | 12% | 10% | 29% |
| abdominal discomfort | 5-10% | 5-14% | 6-17% |
| Transient conduction abnormalities: Overall Transient 2nd degree AVB | 2% 1-2% | 8.5% 4% | 4% 0.1% |
| Transient ST depression of ≥1mm | 8% | 5-7% | 1-2%20 |
| Major side effects | <1% | <1% | <1% |
| Complete AV block | <1% | <1% | Rare |
| Severe (>2 mm) ST depression | <1% | <1% | <1% |
| Fatal or nonfatal MI | Extremely rare | Extremely rare | Extremely rare |
| Stroke | Extremely rare | Extremely rare | Extremely rare |
| Seizures | Rare | Rare | Rare |
| Comments | Symptoms may last longer (15-25 min) than with other vasodilator stressors; Aminophylline frequently required | Due to a short half-time, most side effects resolve in a few (<10) seconds after discontinuation of the infusion; Aminophylline rarely required | Most adverse reactions begin soon after injection and resolve within 15 min (headaches may last up to 30 min) |
Copyright American Society of Nuclear Cardiology

## Slide 5
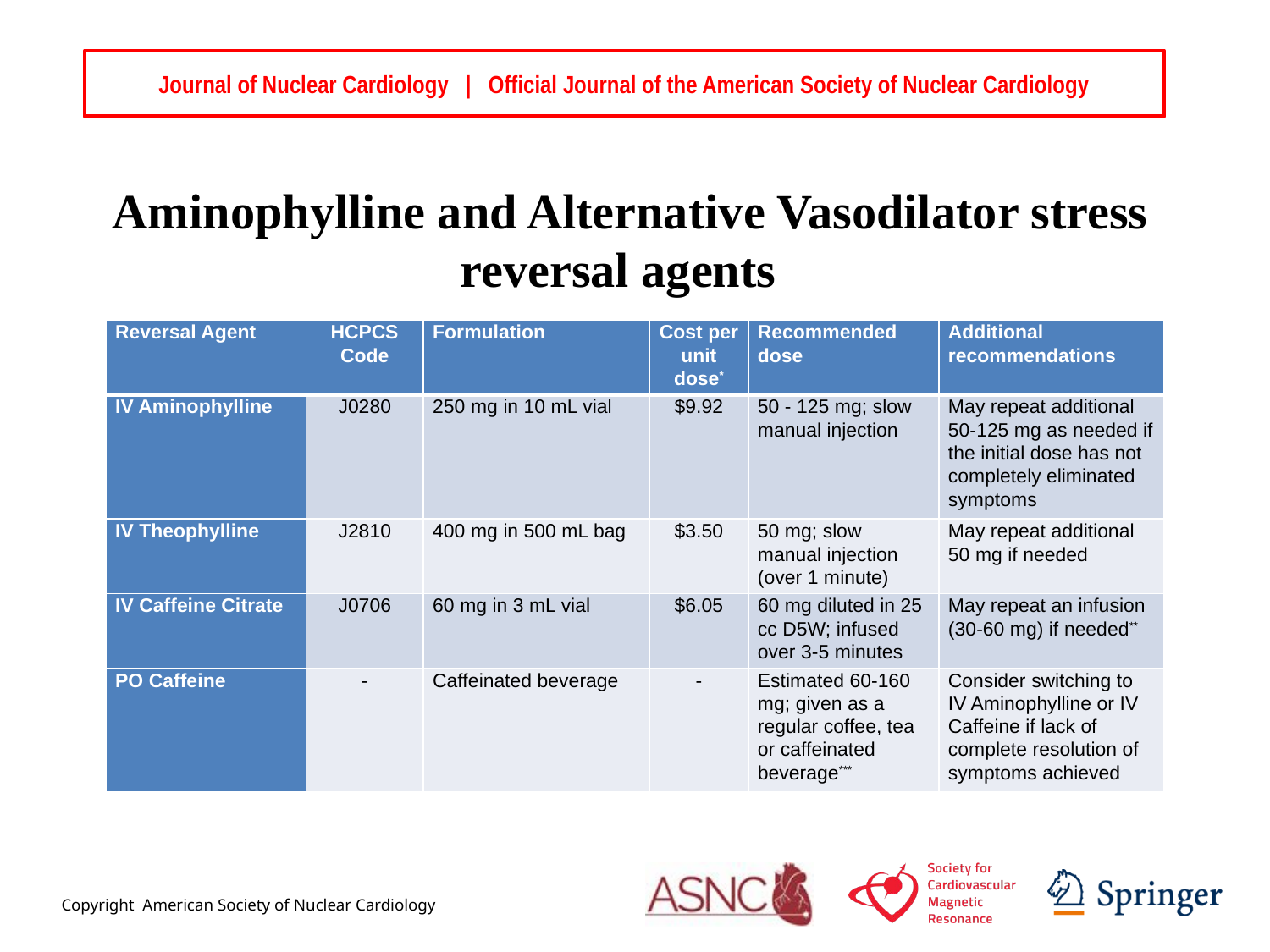

Journal of Nuclear Cardiology | Official Journal of the American Society of Nuclear Cardiology
# Aminophylline and Alternative Vasodilator stress reversal agents
| Reversal Agent | HCPCS Code | Formulation | Cost per unit dose\* | Recommended dose | Additional recommendations |
| --- | --- | --- | --- | --- | --- |
| IV Aminophylline | J0280 | 250 mg in 10 mL vial | $9.92 | 50 - 125 mg; slow manual injection | May repeat additional 50-125 mg as needed if the initial dose has not completely eliminated symptoms |
| IV Theophylline | J2810 | 400 mg in 500 mL bag | $3.50 | 50 mg; slow manual injection (over 1 minute) | May repeat additional 50 mg if needed |
| IV Caffeine Citrate | J0706 | 60 mg in 3 mL vial | $6.05 | 60 mg diluted in 25 cc D5W; infused over 3-5 minutes | May repeat an infusion (30-60 mg) if needed\*\* |
| PO Caffeine | - | Caffeinated beverage | - | Estimated 60-160 mg; given as a regular coffee, tea or caffeinated beverage\*\*\* | Consider switching to IV Aminophylline or IV Caffeine if lack of complete resolution of symptoms achieved |
Copyright American Society of Nuclear Cardiology

## Slide 6
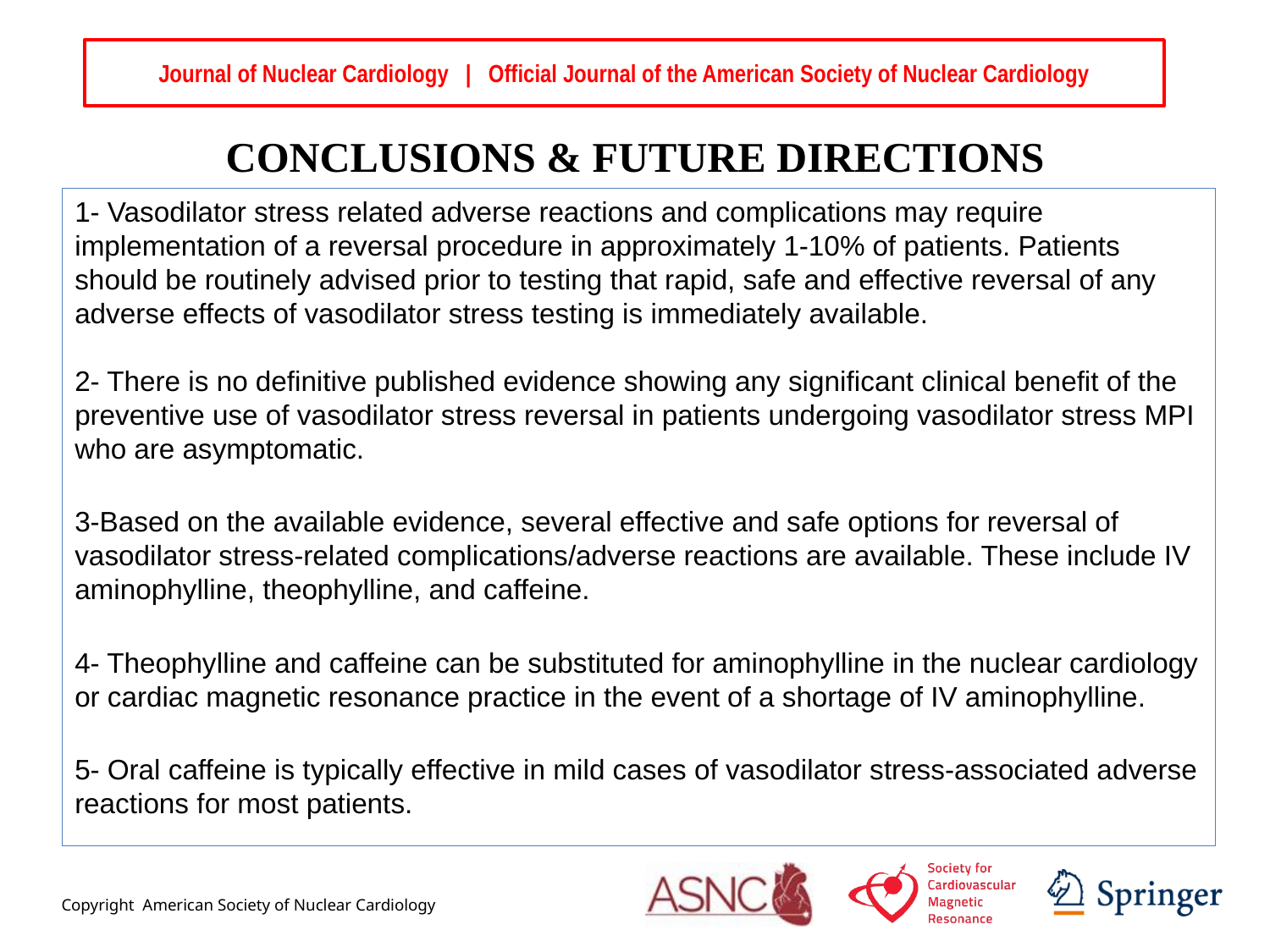

Journal of Nuclear Cardiology | Official Journal of the American Society of Nuclear Cardiology
# CONCLUSIONS & FUTURE DIRECTIONS
1- Vasodilator stress related adverse reactions and complications may require implementation of a reversal procedure in approximately 1-10% of patients. Patients should be routinely advised prior to testing that rapid, safe and effective reversal of any adverse effects of vasodilator stress testing is immediately available.
2- There is no definitive published evidence showing any significant clinical benefit of the preventive use of vasodilator stress reversal in patients undergoing vasodilator stress MPI who are asymptomatic.
3-Based on the available evidence, several effective and safe options for reversal of vasodilator stress-related complications/adverse reactions are available. These include IV aminophylline, theophylline, and caffeine.
4- Theophylline and caffeine can be substituted for aminophylline in the nuclear cardiology or cardiac magnetic resonance practice in the event of a shortage of IV aminophylline.
5- Oral caffeine is typically effective in mild cases of vasodilator stress-associated adverse reactions for most patients.
Copyright American Society of Nuclear Cardiology
